# Supplementary figures and images for: Performance discrepancy mitigation in heart disease prediction for multisensory inter-datasets
Source: PeerJ Comput Sci. 2024 Mar 18;10:e1917. doi: 10.7717/peerj-cs.1917 (PMC11041935; doi:10.7717/peerj-cs.1917)

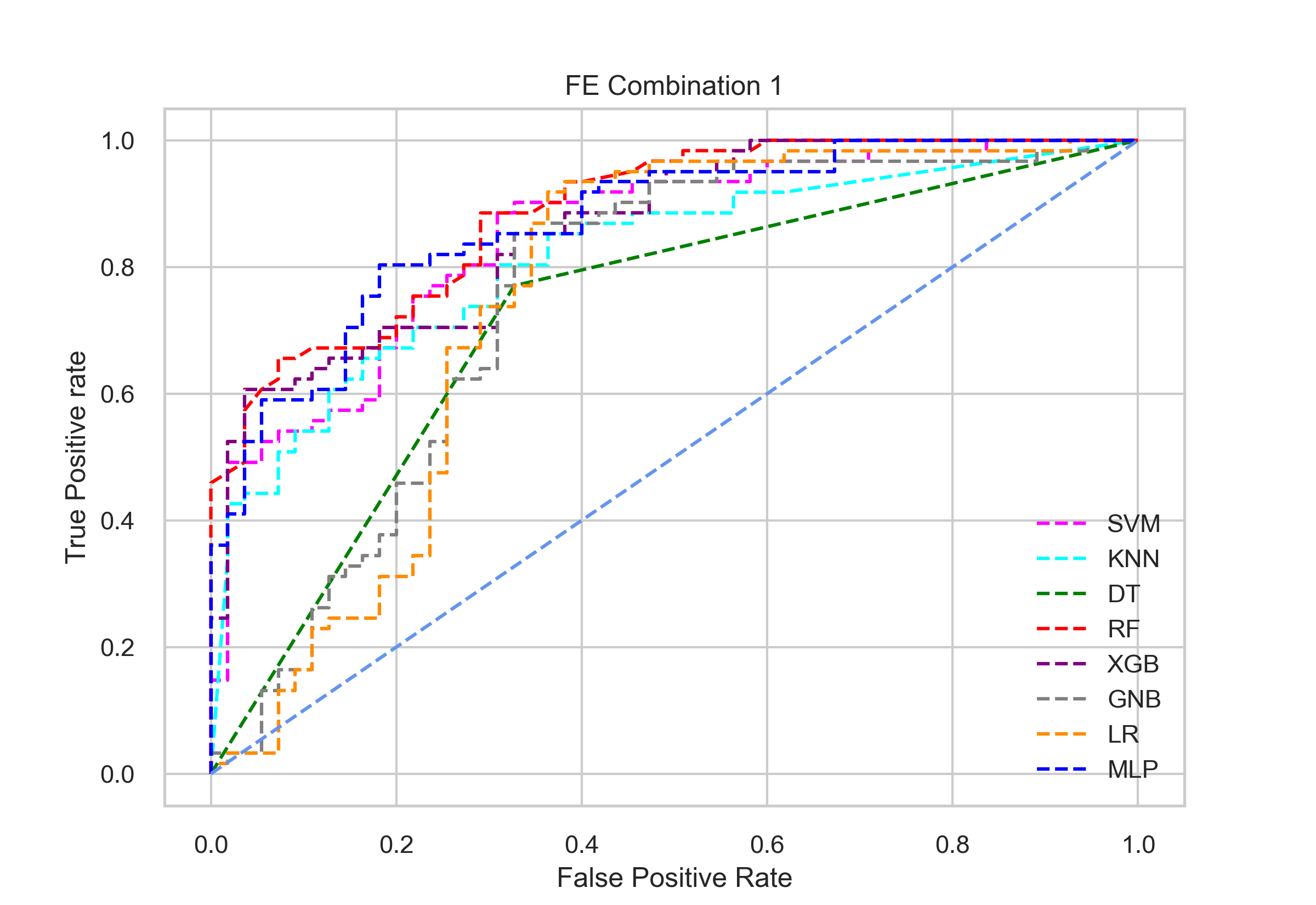

Supplement: Supplemental Information 1 [file peerj-cs-10-1917-s001.zip › Code of Inter Dataset Descripency/Feature Extraction/FE Combination 1.png]
